# Olive Leaf Protein Hydrolysate as a Novel Source of Antimicrobial Peptides: Peptidomic Characterization and In Silico Evaluation

**DOI:** 10.3390/molecules30163382

**Published:** 2025-08-14

**Authors:** Teresa Gonzalez-de la Rosa, Alonso Herreros-Isidro, Elvira Marquez-Paradas, Luna Barrera-Chamorro, Maria J. Leon, Sergio Montserrat-de la Paz

**Affiliations:** 1 Department of Medical Biochemistry, Molecular Biology, and Immunology, School of Medicine, University of Seville, Av. Sanchez Pizjuan s/n, 41009 Seville, Spain; mgonzalez18@us.es (T.G.-d.l.R.); alonsoo_hi@icloud.com (A.H.-I.); emarquez5@us.es (E.M.-P.); lbarrerra1@us.es (L.B.-C.); 2 Instituto de Biomedicina de Sevilla, IBiS, Hospital Universitario Virgen del Rocío, CSIC, University of Seville, 41013 Seville, Spain; 3 Department of Microbiology and Parasitology, School of Pharmacy, University of Seville, C. Profesor Garcia Gonzalez 2, 41012 Seville, Spain; mjl@us.es

**Keywords:** *Olea europaea*, protein hydrolysate, bioactive peptides, in silico analysis, peptidome profile, molecular docking

## Abstract

Olive (*Olea europaea*) leaves, a by-product of olive oil production, represent a promising source of bioactive peptides. In this study, the peptidome of an olive leaf protein hydrolysate (OLPH) obtained via enzymatic hydrolysis with Alcalase was identified and analyzed for the first time. Liquid Chromatography coupled to Trapped Ion Mobility Spectrometry and Tandem Mass Spectrometry (LC-TIMS-MS/MS) analysis revealed over 7000 peptide sequences. Peptides with PeptideRanker scores above 0.79 were selected for in silico evaluation of antimicrobial potential, including physicochemical characterization and molecular docking. Several peptides—such as NYPAWGY, SSKGSLGGGF, QWDQGYF, and SGPAFNAGR—exhibited strong predicted antimicrobial potential, supported by favorable interactions with bacterial, viral, and fungal targets in docking simulations. Correlation analysis revealed that physicochemical features, such as net hydrogen, amphipathicity, and isoelectric point, were positively associated with predicted antimicrobial activity. These findings highlight the potential of olive leaf-derived peptides as natural antimicrobial agents and support the valorization of olive by-products as a sustainable source of functional ingredients for applications in food safety and health. Further experimental validation is needed to confirm the efficacy and mechanism of action of the identified peptides.

## 1. Introduction

The increasing pressure to reduce food waste, combined with the demand for novel bioactive ingredients, has led to a growing interest in underutilized plant residues as alternative sources of high-value compounds. Among these, olive leaves (*Olea europaea* L.) have attracted attention due to their abundance and rich composition in phytochemicals [1]. While the polyphenol content of olive leaves has been widely studied, particularly for its antioxidant and anti-inflammatory properties [2], the protein fraction remains underexploited despite its promising potential for the generation of bioactive peptides [3].

Proteins embedded in plant matrices often contain encrypted peptides that are inactive within the parent sequence but can be released through enzymatic hydrolysis, revealing diverse biological activities [4]. Bioactive peptides derived from food proteins have been shown to exert multifunctional roles, including antihypertensive, immunomodulatory, antioxidant, antidiabetic, and antimicrobial effects [5]. The biotechnological generation of such peptides through enzymatic hydrolysis not only enhances the functional profile of the original protein source but also contributes to the development of clean-label bioactive ingredients with potential applications in food preservation, functional foods, and pharmaceutical formulations.

Recent studies, including our previous work on insect-derived protein hydrolysates, have demonstrated that controlled enzymatic hydrolysis with broad-specificity proteases such as Alcalase can yield peptide fractions enriched in low-molecular-weight (MW) sequences, many of which are predicted to have high bioactivity based on their physicochemical properties and sequence motifs [6,7,8]. For example, hydrolysates obtained from *Tenebrio molitor* protein using Alcalase and evaluated through LC-MS/MS and in silico prediction tools revealed the presence of peptide sequences with strong antimicrobial potential, notably against bacterial and fungal targets [6].

Antimicrobial peptides (AMPs) are of particular interest due to the global rise in antimicrobial resistance and the need for new agents capable of targeting pathogenic microorganisms without contributing to resistance mechanisms [9,10]. AMPs are typically short (5–50 amino acids), positively charged, and amphipathic peptides that can disrupt microbial membranes or interfere with intracellular processes. Their mechanism of action generally involves electrostatic interaction with negatively charged microbial surfaces, insertion into the lipid bilayer, and subsequent membrane permeabilization or lysis. Some AMPs also act by modulating immune responses or interfering with nucleic acid synthesis [11]. The identification of such peptides in food protein hydrolysates represents a promising approach to developing natural antimicrobial agents with dual functionality—nutritional and protective. In silico approaches allow rapid screening of large peptide libraries for their bioactivity potential, toxicity prediction, and interaction with biological targets. Tools, such as PeptideRanker [12], ToxinPred [13], CAMPR3 [14], AMPFun [15], Antimicrobial Peptide Scanner [16], and Macrel [17], enable the classification and scoring of peptides based on their likelihood of being bioactive, antimicrobial, or safe for consumption. In addition, molecular docking simulations can provide insights into the binding affinity of selected peptides with microbial receptors, thereby supporting the rationale for their antimicrobial efficacy. This in silico pipeline complements experimental work, accelerates candidate prioritization, and reduces the cost and time associated with peptide discovery.

The current study aims to fill the existing knowledge gap regarding the peptidome of olive leaf proteins and their potential as a source of AMPs. Specifically, we hypothesize that hydrolysis of olive leaf proteins using Alcalase will result in a peptide-rich hydrolysate containing sequences with physicochemical characteristics compatible with antimicrobial activity. The results will provide a scientific foundation for the utilization of olive leaf proteins as a novel source of bioactive peptides with potential applications in health and food systems.

## 2. Results

### 2.1. In Silico Evaluation of OLPH15A Peptidome

The results of the physicochemical characteristics and bioactivities of the selected sequences obtained from the Peptide Ranker and ToxinPred analyses are shown in Table 1. Initially, all peptide sequences identified from *Olea europaea* L. leaves were compiled. To prioritize sequences with higher potential bioactivity, selection criteria were applied: MW below 1000 Da, a maximum length of 20 amino acids, and absence of post-translational modifications is required, the latter due to the limitations of in silico tools. Small peptides within this size range are more likely to be absorbed and penetrate cells effectively [18]. From over 7000 initial sequences, 2751 met these criteria and were retained for further analysis. These sequences were evaluated using the PeptideRanker tool, which assigns a probability score for peptide bioactivity. According to the developers, scores above 0.5 indicate potential bioactivity, with values exceeding 0.8 being strongly predictive [12]. Among the top 100 peptides selected, 82 were scored above 0.8, and the remaining 18 had values over 0.78, still indicating considerable potential.

Toxicity can be one of the reasons that prevent the incorporation of bioactive peptides into the human diet [19], making it almost mandatory to predict the toxicity of said sequences in order to select those that lack this property. With this aim, ToxinPred was used to evaluate the toxicity of each sequence, revealing that none of them would be hazardous when administered to human beings, suggesting a suitable safety profile for the use of the peptides in alimentary, cosmetic, or therapeutic products. Apart from classifying the peptides according to their likelihood of being toxic, this tool can also predict physicochemical properties of the peptides submitted, which are shown in Table 1. These properties are often linked to the bioactivity of the peptides, making them of interest in making the predictions more accurate. For instance, moderate hydrophobicity values (0.1–0.3), along with appropriate amphipathicity and hydrophilicity, enhance membrane interaction without inducing undesired aggregation [20,21]. Excessive hydrophobicity can lead to mammalian cell toxicity, losing antimicrobial properties. According to Zhang et al. [22], higher amphipathicities result in lower red blood cell lysis. When considering net charge, AMPs display a net positive charge or contain a specific cationic domain to allow the interaction with the phospholipid head in the prokaryotic cell membranes [21,23]. Nevertheless, in our sequences, the maximum net charge observed was +1,00, without structural cationic domains. Therefore, it is plausible that other properties, such as hydrophobicity, contribute to their antimicrobial activity.

It is also important to note that the net charge is pH-dependent. Since the values presented in Table 1 were estimated at neutral pH, peptides with pI values below neutral pH would exhibit a higher net positive charge in acidic environments. Notably, peptides with high antimicrobial activity tend to maintain a positive charge across a broader pH range, typically associated with higher pI values [24]. Future studies aimed at determining protonation constants across different pH values would provide a more comprehensive physicochemical characterization and deepen the understanding of peptide behavior in diverse physiological environments.

Lastly, MW is a relevant parameter to consider due to its relevance in stability and absorption [25]. There is evidence that reducing the MW while maintaining the net charge and the hydrophobicity can optimize antimicrobial activities, cell-penetration, stability, and synthesis efficiency. Specifically, it seems that peptides with a MW < 1000 Da achieve a good balance among biological activity, synthesis, and pharmacological profile [26]. Specifically, peptides in this size range retain effective antimicrobial activity while reducing synthesis cost and complexity.

Table 2 summarizes the predicted antimicrobial activity of the 100 peptides with the highest bioactivity scores. The first tool, CAMPR3 [14], classified the peptides as antimicrobial or non-antimicrobial based on several machine learning algorithms, each producing different outcomes. Using the Support Vector Machine (SVM) classifier, 34 peptides were identified as AMPs, while the Artificial Neural Network (ANN) model classified 69 sequences. In contrast, the Random Forest and Discriminant Analysis models predicted only 2 and 10 AMPs, respectively. Next, AMPfun [15] provided probabilistic scores for overall antimicrobial potential and specificity against microbial groups. This tool generally yielded higher prediction values than CAMPR3. Notably, the highest mean probability corresponded to activity against Gram-positive bacteria, followed by antiviral potential. This cross-activity proves that our peptides have a wide range of action and that they are compatible with therapeutic strategies to face mixed or multi-resistant infections. Furthermore, the predicted specificity for Gram-positive and negative bacteria allows us to select those sequences more effective against a certain group. Further analysis with Antimicrobial Peptide Scanner v2 [16] identified 59 peptides as AMPs. Considering all the peptides marked as AMP, there were 59 out of the 100 that fit in this group. Lastly, the AMP probability for all the sequences was determined using the bioinformatic tool Macrel [17], which provided lower AMP probability scores overall, likely due to the optimization of this tool for higher specificity in its predictions.

Altogether, the integration of physicochemical properties with the antimicrobial prediction profiles enables the prioritization of the candidate peptides with an adequate balance among bioactivity, specificity, and safety. This computational approach can speed up the design of new therapeutic agents consisting of or derived from peptides, as well as their usage in biomedical and biotechnological products.

Previous studies have successfully combined in silico predictions of antimicrobial, antifungal, and antiviral peptide activities with subsequent in vitro validation, demonstrating the reliability of such integrative approaches for identifying bioactive peptides from natural sources [27,28]. These findings support the predictive strategy applied in our work and highlight the importance of experimental validation in future research.

**Figure 1:**
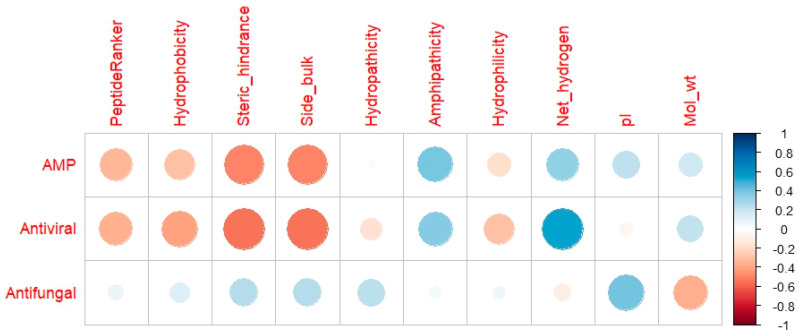
Correlation heatmap between peptide characteristics (columns) and their antibacterial, antifungal, and antiviral predicted capacities (rows). The color and size of each circle represent Pearson’s correlation coefficient.

The relationships between peptide physicochemical descriptors and predicted antimicrobial activities were further explored using Pearson’s correlation analysis, visualized through a correlation heatmap (Figure 1). This graphical representation allowed for the identification of significant associations between structural features of the peptides and their predicted bioactivities, including general AMP, antiviral, and antifungal potential. In this heatmap, the strength and direction of each correlation are indicated by both the size and the color of the circles. Blue hues denote positive correlations, while red hues indicate negative ones, with darker and larger circles corresponding to stronger coefficients.

Among the most notable findings, antiviral activity showed a strong positive correlation with net hydrogen content (*r* = 0.534), suggesting that peptides with a higher number of hydrogen atoms—likely reflecting greater polarity and/or hydrogen bonding capacity—may be more effective at disrupting viral targets or interacting with viral enzymes. This observation is consistent with previous reports indicating that polar interactions and charge distribution play essential roles in peptide–virus interactions, particularly in docking studies involving viral proteases or fusion proteins [29]. In contrast, strong negative correlations were observed between antiviral potential and both side bulk (*r* = −0.542) and steric hindrance (*r* = −0.542). These findings imply that peptides with more compact side chains and reduced steric hindrance are favored for antiviral activity, possibly due to better accessibility to target sites or more favorable conformational flexibility. This aligns with structural characteristics typically associated with antiviral peptides (AVPs), which often require efficient penetration or binding to relatively constrained active sites in viral proteins.

Regarding general antimicrobial (AMP) activity, moderate negative correlations were observed with steric hindrance (*r* = −0.499) and side bulk (*r* = −0.499), reinforcing the notion that smaller, less hindered peptides are more likely to exhibit broad-spectrum antimicrobial properties. This may be due to their ability to insert more easily into microbial membranes or avoid steric clashes in protein–peptide interactions, both of which are key factors in membrane disruption and intracellular targeting mechanisms [30]. Interestingly, antifungal activity demonstrated a moderate positive correlation with the isoelectric point (pI) of the peptides (*r* = 0.409), indicating that more basic peptides (with higher pI values) may be better suited for targeting fungal pathogens. Fungal membranes are known to contain negatively charged components, such as phosphatidylserine and ergosterol-rich regions, which could enhance electrostatic interactions with cationic peptides, leading to membrane perturbation or intracellular uptake [23]. This is consistent with prior evidence that cationic charge is a critical determinant of antifungal peptide efficacy.

Taken together, these results highlight the complex interplay between peptide structure and predicted bioactivity. The findings support the utility of in silico screening tools, not only to identify promising antimicrobial candidates but also to elucidate structural principles that can guide the rational design or selection of functional peptides from protein hydrolysates. The physicochemical descriptors that showed significant correlations—particularly net hydrogen, pI, steric hindrance, and side bulk—should be considered key parameters in future peptide filtering strategies when working with large peptidomic datasets derived from enzymatic hydrolysis of natural protein sources.

### 2.2. Molecular Docking Analyses

To validate and further explore the functional predictions obtained from the antimicrobial activity screening tools, molecular docking simulations were conducted. The docking analysis aimed to assess the binding affinity and interaction profiles of selected peptides with bacterial, viral, and fungal protein targets. These targets were chosen based on their biological relevance in infectious processes and their structural availability in the Protein Data Bank (PDB). Peptides were selected for docking based on their high predicted antimicrobial potential, as revealed by in silico bioactivity screening tools.

This in silico strategy is intended as a preliminary screening tool to identify promising peptide candidates, and further experimental validation will be necessary to confirm their predicted bioactivities.

#### 2.2.1. Bacterial Receptors

**Figure 2:**
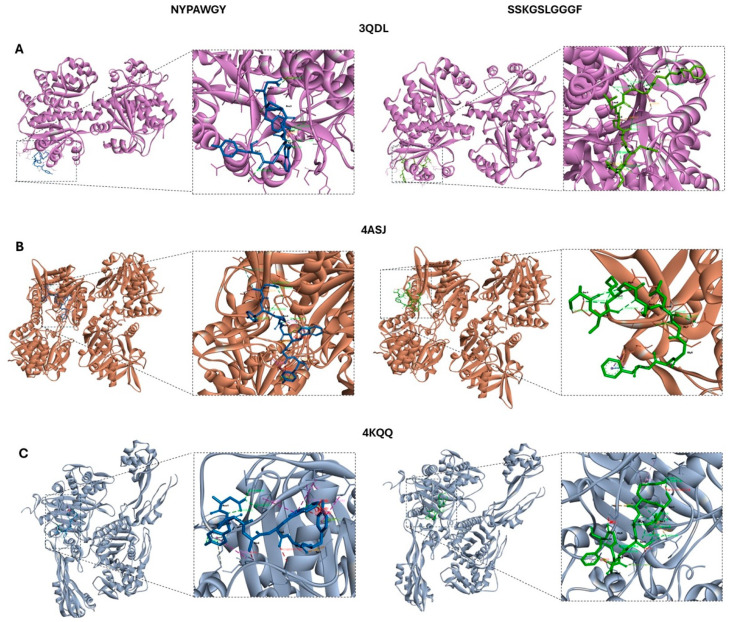
Three-dimensional visualization of the receptor–peptide complexes obtained through molecular docking and rendered using Biovia Discovery Studio Visualizer. The binding sites and key interactions of the peptides NYPAWGY and SSKGSLGGGF with bacterial protein targets are shown: (**A**) NADPH nitroreductase (RdxA) from *Helicobacter pylori* (PDB: 3QDL); (**B**) glucose-1-phosphate thymidylyltransferase (RmIA) from *Pseudomonas aeruginosa* (PDB: 4ASJ); and (**C**) penicillin-binding protein 3 (PBP3) from *P. aeruginosa* (PDB: 4KQQ).

**Figure 3:**
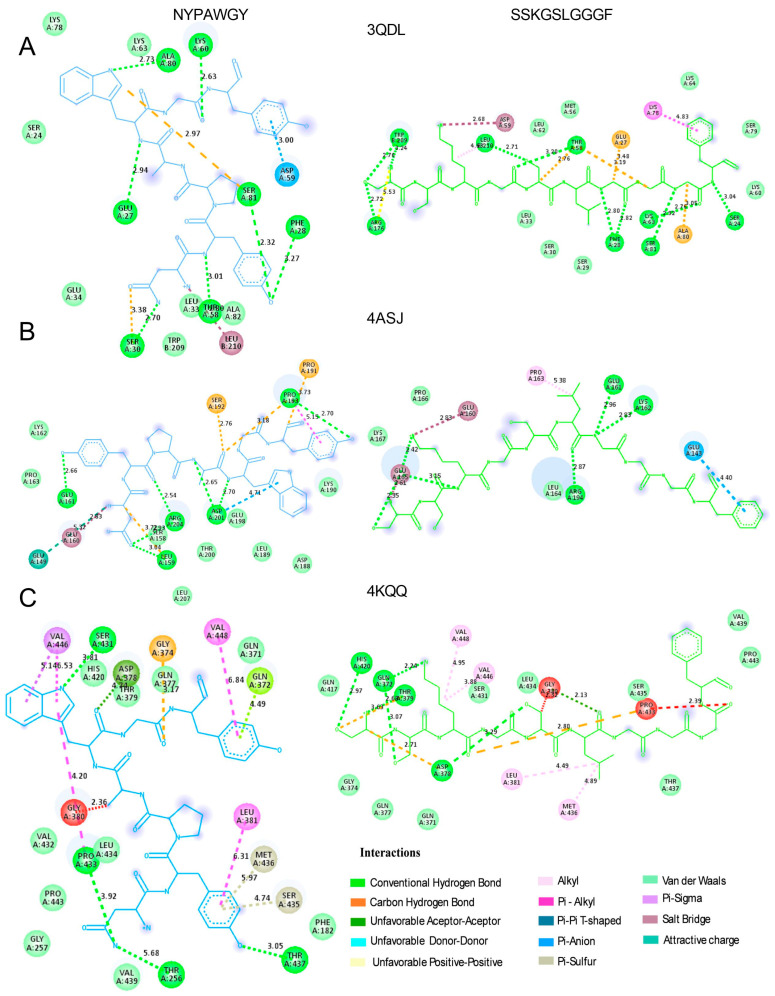
Two-dimensional interaction diagrams of selected peptides docked with bacterial protein targets, generated using Biovia Discovery Studio Visualizer. (**A**) oxygen-insensitive NADPH nitroreductase of *Helicobacter pylori* (RdxA, PDB: 3QDL); (**B**) glucose-1-phosphate thymidylyltransferase from *Pseudomonas aeruginosa* (RmIA, PDB: 4ASJ); and (**C**) penicillin-binding protein 3 from *Pseudomonas aeruginosa* (PBP3, PDB: 4KQQ). Ligand–receptor interactions are annotated by type and proximity, providing structural insight into binding affinity and specificity of the docked peptides.

Among the peptides identified with the highest predicted antibacterial activity, NYPAWGY (UniProt AC: A0A8S0Q314; Alpha-1,4 glucan phosphorylase) and SSKGSLGGGF (unknown) achieved the maximum scores across multiple AMP prediction platforms, suggesting strong potential as effective antibacterial agents. These peptides were prioritized for further structural evaluation due to their favorable physicochemical profiles, including optimal net charge, hydrophobicity, and amphipathicity. In addition, both peptides were selected for molecular docking against three representative bacterial receptors: the oxygen-insensitive NADPH nitroreductase from *Helicobacter pylori* (RdxA, PDB ID: 3QDL, Figure 2A), glucose-1-phosphate thymidylyltransferase from *Pseudomonas aeruginosa* (RmlA, PDB ID: 4ASJ, Figure 2B), and penicillin-binding protein 3 from *P. aeruginosa* (PBP3, PDB ID: 4KQQ, Figure 2C). These proteins are key components in bacterial metabolism and cell wall synthesis and thus represent attractive molecular targets for antimicrobial interventions. Figure 2 illustrates the 3D structures of the peptide–protein complexes, highlighting the spatial orientation and binding modes of the ligands. Figure 3 provides detailed 2D interaction diagrams, identifying key hydrogen bonds, hydrophobic contacts, and π-alkyl interactions. A summary of docking scores, binding residues, and interaction distances is provided in Table 3.

Bacterial receptors were selected based on their clinical significance and functional roles in antimicrobial susceptibility. The first target, oxygen-insensitive NADPH nitroreductase (RdxA) from *Helicobacter pylori* (PDB: 3QDL), plays a pivotal role in the reductive activation of metronidazole, a frontline antibiotic used in the treatment of *H. pylori*-associated gastric infections. Mutations in the rdxA gene have been directly linked to metronidazole resistance, highlighting the therapeutic significance of this enzyme as a pharmacological target [31]. The second receptor, glucose-1-phosphate thymidylyltransferase (RmlA) from *Pseudomonas aeruginosa* (PDB: 4ASJ), catalyzes the formation of dTDP-L-rhamnose, an essential sugar for the biosynthesis of cell wall components such as lipopolysaccharides (LPS). These structures are critical for bacterial virulence, membrane stability, and immune evasion. Inhibiting RmlA can disrupt LPS assembly and compromise the integrity of the bacterial envelope, rendering the pathogen more susceptible to host defenses and antibiotic treatments [32]. The third bacterial target was the penicillin-binding protein 3 (PBP3) from *P. aeruginosa* (PDB: 4KQQ), a transpeptidase involved in the final stages of peptidoglycan biosynthesis and crucial for septal cell wall formation during bacterial cell division. PBP3 is a known target of β-lactam antibiotics, such as ceftazidime and aztreonam, and resistance to these agents is often associated with altered expression or mutation of this protein [33]. Therefore, alternative molecules capable of binding to PBP3 could offer new avenues for antibacterial drug development, particularly against multidrug-resistant strains.

The binding affinities of both peptides across the various receptors ranged from −15.3 to −7.5 kcal/mol. Notably, the peptide SSKGSLGGGF exhibited the lowest binding energy with the RmlA from *Pseudomonas aeruginosa,* suggesting its potential as a promising candidate for therapeutic antimicrobial development. Regarding interactions, hydrogen bonds with polar residues predominated, accompanied by π-alkyl interactions with hydrophobic residues, suggesting stable binding to the enzyme, while van der Waals interactions are established, probably contributing to a strong anchoring of the peptide. Conventional hydrogen bonds are likely to stabilize the complex and potentially induce conformational changes in the receptor structure [34].

Altogether, these results demonstrate the ability of the peptides to adopt distinct binding conformations depending on the structural features of each receptor, enabling interactions with critical catalytic or allosteric sites of enzymes essential for bacterial viability and pathogenicity. The variability in binding modes further supports their potential as versatile scaffolds for the development of novel antimicrobial agents targeting a wide range of bacterial enzymes involved in resistance mechanisms and virulence.

#### 2.2.2. Viral Receptors

**Figure 4:**
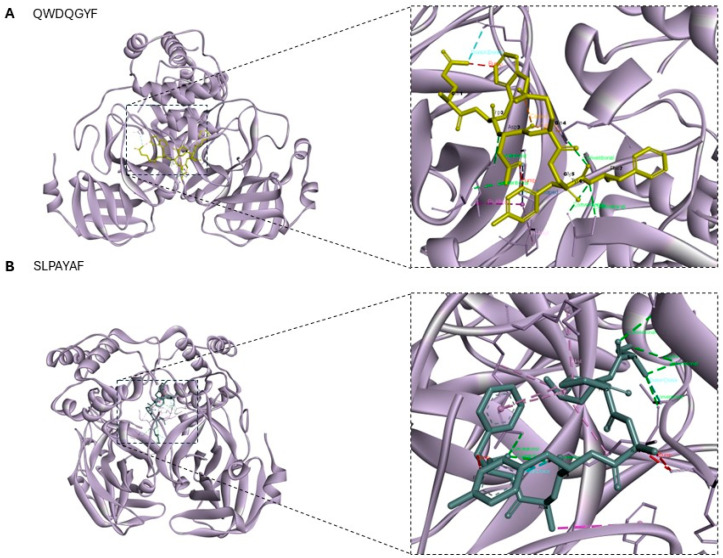
Three-dimensional visualization of peptide–receptor complexes generated through molecular docking and visualized using Biovia Discovery Studio Visualizer. (**A**) COVID-19–QWDQGYF binding site and interactions; and (**B**) COVID-19–SLPAYAF binding site and interactions.

**Figure 5:**
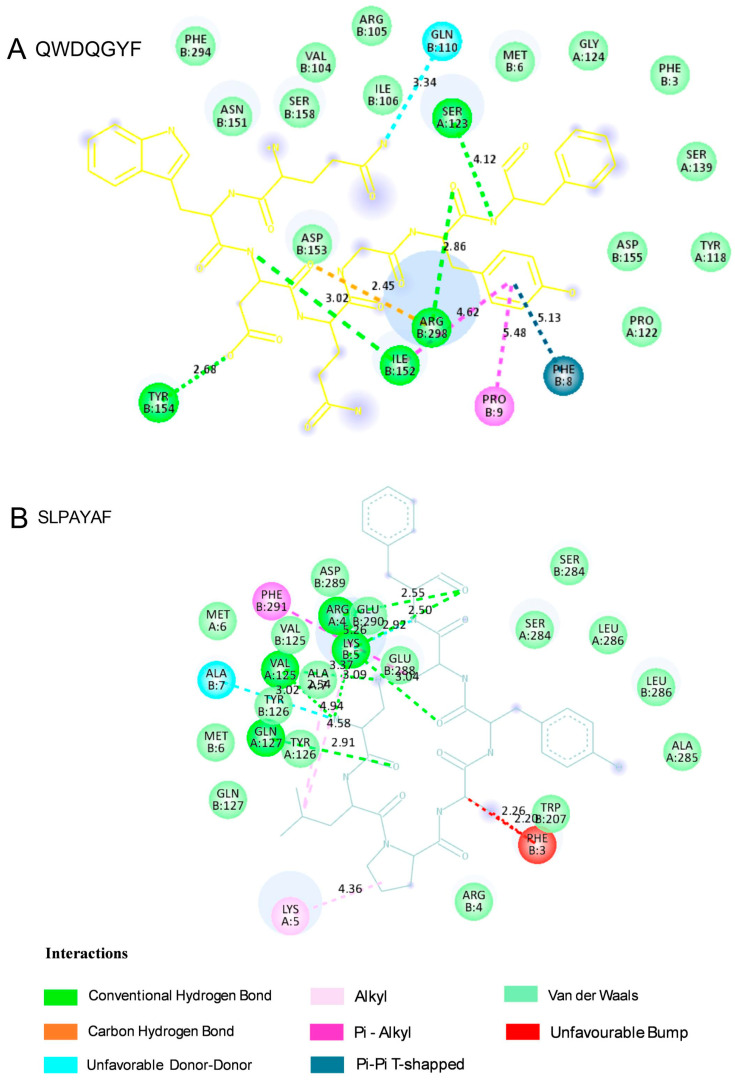
Two-dimensional interaction diagrams of selected peptides docked with the SARS-CoV-2 main protease (M^pro^) in its apo state, visualized using Biovia Discovery Studio Visualizer. The diagrams illustrate key interactions between the peptides (**A**) QWDQGYF and (**B**) SLPAYAF and the protease.

The peptides that showed the most promising antiviral activity were sequences QWDQGYF (UniProt AC: I3NSV1; Photosystem II CP47 reaction center protein) and SLPAYAF (UniProt AC: A0A2K9RS12; Photosystem I P700 chlorophyll apoprotein A2), both with scores of 0.91. Molecular docking of antiviral peptides against the COVID-19 main protease in its apo state (PDB: 7C2Q) is crucial for discovering new therapeutic strategies targeting a key enzyme essential for SARS-CoV-2 replication [35]. Given the global impact of COVID-19 and the need for effective antiviral treatments, this approach supports the development of peptide-based inhibitors with the potential to aid the rational design of new peptide-based therapies. The molecular docking analysis of the SARS-CoV-2 main protease (M^pro^) in its apo state with the peptides QWDQGYF and SLPAYAF revealed distinctive and functionally relevant binding patterns that underscore their potential as antiviral agents. Both peptides were shown to bind to the M^pro^ dimer interface. Moreover, both peptides showed remarkable affinities for this viral protein, with values of −13.4 and −10.9, respectively. In Figure 4 and Figure 5, the visualization of the interactions of each peptide with the SARS-CoV-2 main protease in the apo state is depicted, and the different types of interaction are reported in Table 4, including the distances and residues involved in each interaction.

For both peptides, the docking analysis predicted multiple stabilizing interactions, including conventional hydrogen bonds along with π-alkyl contacts, in addition to notable van der Waals contacts, which could stabilize their placement within the enzyme’s cavity (Figure 4, Table 4). However, in the case of SLPAYAF, the presence of unfavorable steric protrusions might suggest possible conformational constraints or repulsive interactions that could be reducing binding affinity or inhibiting optimal positioning for catalytic interference. Interestingly, these results align with recent findings showing that the plasticity of the M^pro^ interface could accommodate diverse ligands [36,37]. As visualized in Figure 5B, SLPAYAF was shown to bind specifically to Domain III, a five-helix globular bundle that is involved in the regulation of M^pro^ dimerization through a salt-bridge interaction between Glu290 of one protomer and Arg4 of the other [38], so this peptide might be able to inhibit M^pro^ dimerization. Together, these data provide valuable insights into the nature of peptide-protease interactions in SARS-CoV-2 M^pro^, suggesting that these peptides could be useful to design peptide-based inhibitors targeting M^pro^, supporting ongoing strategies aimed at identifying novel therapeutics for COVID-19.

#### 2.2.3. Fungal Receptor

**Figure 6:**
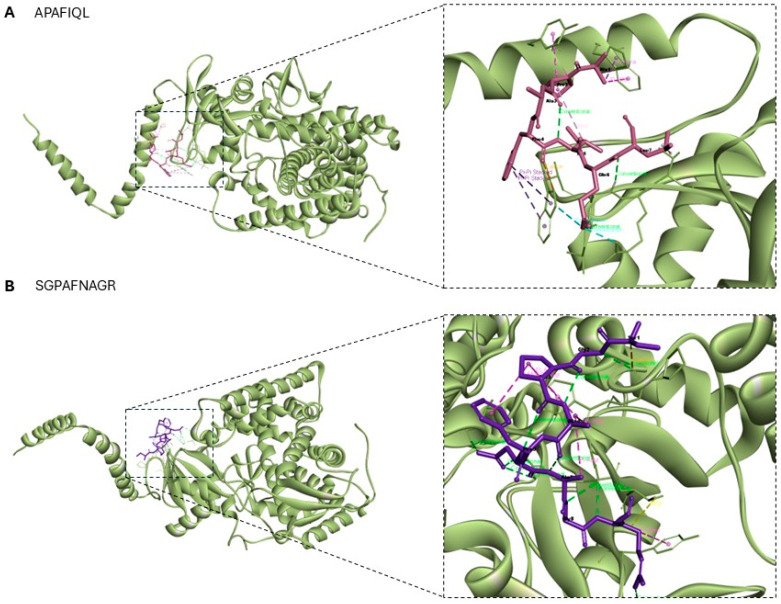
Three-dimensional visualization of receptor–peptide complexes generated using Biovia Discovery Studio Visualizer. (**A**) 4LXJ–APAFIQL binding site and interactions; and (**B**) 4LXJ–SGPAFNAGR binding site and interactions.

**Figure 7:**
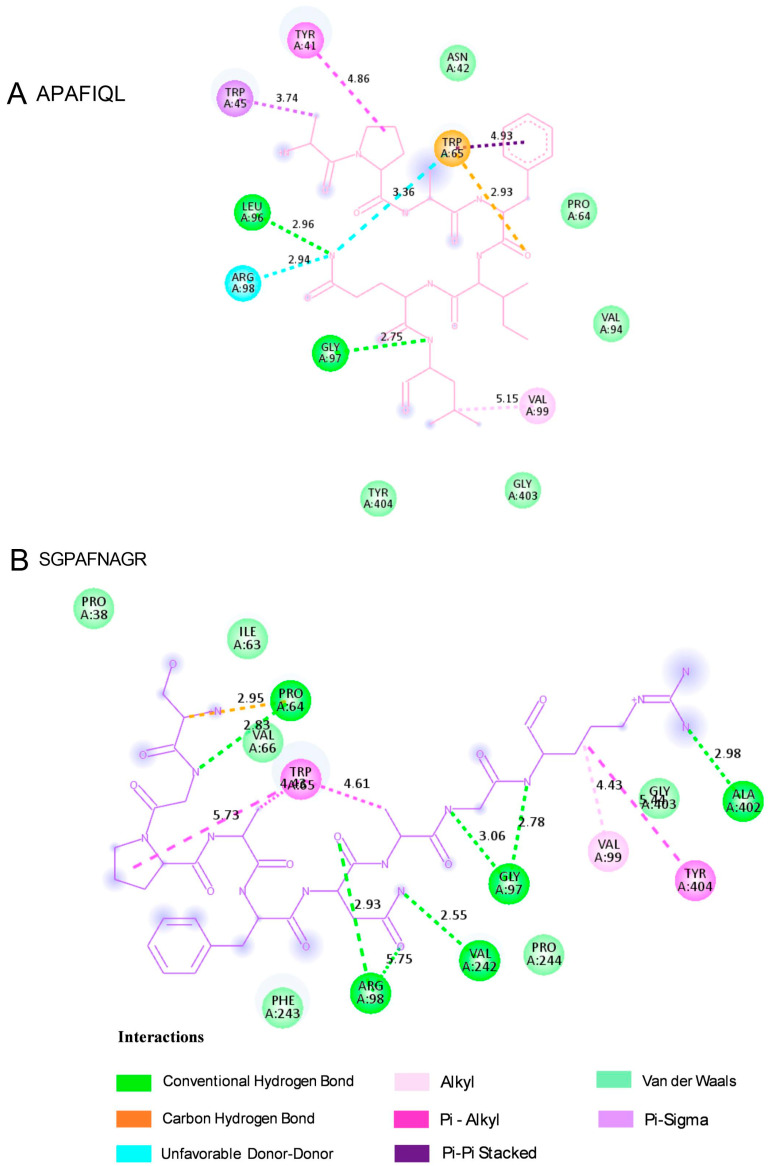
Two-dimensional interaction diagrams of the selected peptides (**A**) APAFIQL and (**B**) SGPAFNAGR docked with lanosterol 14-α-demethylase from *Saccharomyces cerevisiae* (PDB: 4LXJ), visualized using Biovia Discovery Studio Visualizer.

Finally, for molecular docking analysis with the fungal receptor lanosterol 14-α demethylase (CYP51, PDB ID: 4LXJ) from *Saccharomyces cerevisiae,* a cytochrome P450-dependent monooxygenase involved in ergosterol biosynthesis is used. This enzyme is a well-known pharmacological target of azole antifungal drugs, as it catalyzes a key demethylation step in the conversion of lanosterol to ergosterol, an essential component of fungal cell membranes. Inhibiting this enzyme disrupts membrane structure and function, ultimately leading to fungal cell death [39]. Among the peptides identified with predicted antifungal activity, APAFIQL (Uniprot AC: F6I9E4; ATP synthase subunit beta, chloroplastic) and SGPAFNAGR (Uniprot AC: A0A2K9RS12; Photosystem I P700 chlorophyll an apoprotein A2) achieved the highest scores (0.72 and 0.73, respectively) and were therefore selected for docking. The binding sites and molecular interactions between the analyzed peptides and the fungal receptor were visualized in 3D, as shown in Figure 6. Additional structural information is provided in Figure 7 and concisely summarized in Table 5. The results revealed distinct and functionally relevant interaction profiles, underscoring their potential as modulators of this essential fungal enzyme.

In this case, the binding affinity of the APAFIQL peptide is −9.2 kcal/mol and for SGPAFNAGR is −9.9 kcal/mol, and recent studies have reported similar affinity values for other compounds with antifungal capacity [40,41]. The presence of aromatic and hydrophobic contacts suggested a strong anchoring of the peptide, likely interfering with the binding or orientation of the natural substrate lanosterol. Taken together, these docking results offer mechanistic insights into the potential of APAFIQL and SGPAFNAGR to disrupt lanosterol demethylation, highlighting their promise as lead structures for the development of peptide-based antifungal agents targeting the ergosterol biosynthetic pathway.

## 3. Materials and Methods

### 3.1. Chemical and Samples

Leaves of *Olea europaea* were donated by local olive growers in Andalusia (Spain) and used exclusively for experimental purposes. Alcalase 2.4 L FG (2.4 AU-A/g) was donated by Novozymes (Bagsvaerd, Denmark). All chemicals (reagents and solvents) were of analytical grade and came from Sigma Chemical Co., Bachem AG (Bubendorf, CH, EU), and Gibco (Waltham, MA, USA).

### 3.2. Protein Concentration of Olea europaea Leaves

Olive leaves were ground using a blade mill to obtain a homogeneous powder, referred to as OLP (olive leaf powder). The protein concentrate (OLPc) was extracted by ultrasound-assisted alkaline extraction (UAAE), following the method of Ortega et al. [3] with slight modifications. OLP was suspended in 0.1 M NaOH (1:20 g/mL), adjusted to pH 9, and stirred for 1 h at 25 °C. The mixture was then sonicated for 1 h at 30 °C in an ultrasonic bath (Witeg, Wertheim, Germany), filtered under vacuum, and acidified to pH 4 to precipitate proteins. Samples were stored at 4 °C for 2 h, centrifuged at 4000 rpm for 30 min at 4 °C, and the resulting pellet was washed with pH-adjusted distilled water to improve the yield. After repeated centrifugation, the protein concentrate was dried under nitrogen, stored at −80 °C, and freeze-dried for further analysis.

### 3.3. Hydrolysis of Olea europaea Concentrate

Hydrolysis of OLPc was performed in a jacketed reactor under continuous stirring at a constant temperature of 50 °C and pH 8. A total of 1.50 g of OLPc was resuspended in distilled water, and Alcalase 2.4 L FG was added at an enzyme-to-substrate (E/S) ratio of 2.75%. This selected E/S ratio was based on previous studies focusing on the generation of bioactive peptides [42,43]. A 15 min hydrolysis yielded a degree of hydrolysis close to 10%, which has proven sufficient to identify a large number of peptides in the LC-TIMS-MS/MS workflow [44]. After this time of hydrolysis, enzymatic activity was halted by freezing the sample at −80 °C. The hydrolysate (designated OLPH15A) was subsequently freeze-dried and stored at −20 °C until further analysis.

### 3.4. Peptide Extraction, Purification, and Sequence Identification by LC-TIMS-MS/MS

The samples were acidified with 0.5% trifluoroacetic acid (*v*/*v*). The desalting and concentration steps were performed with ZipTip C18 (Millipore), and the samples were speed-vacuum dried. LC-TIMS-MS/MS was carried out using a nanoElute nanoflow ultrahigh-pressure LC system (Bruker Daltonics, Bremen, Germany) coupled to a timsTOF Pro 2 mass spectrometer, equipped with a CaptiveSpray nanoelectrospray ion source (Bruker Daltonics) according to the procedure described in the patent P202230873.

Briefly, approximately 200 ng of peptide digest was loaded onto a Bruker FIFTEEN C18 capillary column (15 cm length, 75 μm ID, 1.9 μm particle size, 120 Å pore size; Bruker Daltonics). Peptides were separated at 30 °C using a 20 min gradient at a flow rate of 300 nL/min (mobile phase A (MPA): 0.1% FA; mobile phase B (MPB): 0.1% FA in acetonitrile). A step gradient from 0 to 35% MPB was applied over 13 min, followed by a 35–90% MPB step of 13–15 min, and finished with a 90% MPB wash for an additional 5 min for a further time. timsTOF Pro 2 was run in DDA-PASEF mode. Mass spectra for MS and MS/MS scans were recorded between 100 and 1700 *m*/*z*. Ion mobility resolution was set to 0.85–1.30 V s/cm2 over a ramp time of 100 ms. Data-dependent acquisition was performed using 4 PASEF MS/MS scans per cycle with a duty cycle close to 100%. A polygonal filter was applied on the *m*/*z* space and ion mobility to exclude low *m*/*z*, mainly single-charged ions from the selection of PASEF precursors. An active exclusion time of 0.4 min was applied to precursors that reached 20,000 intensity units. The collision energy was increased stepwise as a function of the ion mobility ramp, from 27 to 45 eV. The raw data were analysed in PEAKS Studio ProX (Bioinformatics Solution Corp, Waterloo, ON, Canada). The reference library is acquired from uniprotkb_taxonomy_Olea-europaea_2025_03_17. The raw data were analysed with parent mass error tolerance set to 15 ppm and a fragment mass error tolerance of 0.05 Da. To account for post-translational modifications and chemical labeling, the following settings were used: carbamidomethylation of cysteine residues was set as the fixed modification, methionine oxidation and acetylation (Protein N-term) were set as the variable modification. Protein unique peptides were set to larger than 1, and a high confidence score of −10lgP > 17.5 was applied to indicate an accurately identified protein.

### 3.5. In Silico Analysis

#### 3.5.1. Prediction Tools

Peptide sequences identified from *Olea europaea* L. leaves were filtered based on molecular weight (<1000 Da), length (≤20 amino acids), and absence of post-translational modifications. From over 7000 initial sequences, 2751 met these criteria. Peptide bioactivity was predicted using PeptideRanker, with scores above 0.5 indicating potential activity [12]. The top 100 candidates included 82 peptides with scores >0.8 and 18 with scores >0.78.

All these peptides were subjected to the following prediction tools: (a) ToxinPred software was used to predict, for each peptide, its hydrophobicity, side bulk, amphipathicity, hydrophilicity, hydropathicity, steric hindrance, toxicity, net hydrogen, isoelectric point, charge, and molecular weight [13]; (b) to estimate the likelihood of being antimicrobial using different algorithms, the tools CAMPR3 [14], Antimicrobial Peptide Scanner vr.2 [16] and Macrel [17] were employed (c) AMPfun was used to estimate the likelihood of being specifically antibacterial, antiviral, or antifungal [15].

#### 3.5.2. In Silico Correlation of Parameters

Prior to conducting statistical analyses, the dataset underwent preprocessing, which involved the removal of duplicate peptide entries to ensure the integrity of the results. To identify potential correlations of interest among variables, Pearson’s correlation coefficient was calculated as a measure of linear association. Correlation heatmaps were subsequently generated from the resulting correlation matrices, depicting relationships between peptide physicochemical properties and their predicted antifungal, antiviral, and antibacterial activities [45]. Visualization of these heatmaps was performed using the *ggplot2* package (version 3.4.2) implemented in the R statistical environment.

#### 3.5.3. Molecular Docking

Molecular docking simulations were performed to evaluate the binding affinity of selected peptides against a range of bacterial, viral, and fungal receptors. The bacterial targets included oxygen-insensitive NADPH nitroreductase from *Helicobacter pylori* (RdxA, PDB ID: 3QDL), glucose-1-phosphate thymidylyltransferase from *Pseudomonas aeruginosa* (RmIA, PDB ID: 4ASJ), and penicillin-binding protein 3 from *P. aeruginosa* (PBP3, PDB ID: 4KQQ). The viral receptor was the main protease of SARS-CoV-2 in its apo state (PDB ID: 7C2Q), and the fungal target was lanosterol 14-α demethylase from *Saccharomyces cerevisiae* (PDB ID: 4LXJ). All receptor structures were retrieved from the Protein Data Bank (RCSB PDB, http://www.rcsb.org, accessed on 25 July 2025). Ligands and all the water molecules were removed from the receptors’ PDB files, while polar hydrogen atoms were added by UCSF Chimera software. The 3D structures of the peptides were obtained, prepared for docking, and structurally minimized using the same software. Docking analyses were performed using different peptides for each receptor, selected based on their AMPfun scores in antibacterial, antifungal, and antiviral predictions. In the case of the bacterial receptors, the simulation was performed with the peptides NYPAWGY and SSKGSLGGGF; and QWDQGYF and SLPAYAF for the viral target; finally, the peptides analysed with the fungal receptor were SGPAFNAGR and APAFIQL. Then, the molecular structures of receptors and peptides were converted to PDBQT format with AutoDock Tools, and the grid box parameters for each receptor–peptide complex were defined using AGFR. Docking simulations were then carried out, and the best binding poses were selected based on their docking scores. The resulting complexes were visualized in Biovia Discovery Studio Visualizer, including detailed 2D interaction diagrams and surface mapping of receptor–ligand contacts.

## 4. Conclusions

In this study, we have demonstrated that OLPH15A, obtained through enzymatic hydrolysis with Alcalase, is a rich source of short, non-toxic peptides with promising antimicrobial potential. Peptidomic analysis revealed more than 7000 unique peptide sequences, among which a subset exhibited physicochemical characteristics and in silico bioactivity scores indicative of antimicrobial function. The integration of multiple computational tools allowed for the prioritization of candidates based on antimicrobial probability, safety, and structural features. The correlation between physicochemical properties and predicted antimicrobial activity highlighted key structural determinants—such as low steric hindrance, compact side chains, and high isoelectric point—that may guide future screening strategies. Docking simulations confirmed that selected peptides exhibited strong and specific interactions with key bacterial (RdxA, RmlA, PBP3), viral (SARS-CoV-2 main protease), and fungal (CYP51) targets, reinforcing their therapeutic potential. These findings support the valorization of olive leaves, an agricultural by-product, as a sustainable source of functional peptides with antimicrobial properties. Moreover, our results offer a framework for combining high-throughput peptidomics and in silico analysis in the discovery and pre-validation of natural antimicrobial peptides. Future studies are warranted to experimentally validate the bioactivity of the most promising candidates and assess their stability, spectrum of action, and mechanism in biological systems.

## Figures and Tables

**Table 1:** Characterization of the most bioactive peptides from the peptidome according to the PeptideRanker prediction tool, identified in OLPH15A, based on in silico analyses in relation to the physicochemical properties.

Peptide	PeptideRanker ^1^	ToxinPred ^2^										
		Toxicity	Hydrophobicity	Steric Hindrance	Side Bulk	Hydropathicity	Amphipathicity	Hydrophilicity	Net Hydrogen	Charge	pI	MW
SGFGGFGGGF	0.97	Non-Toxin	0.25	0.67	0.67	0.52	0.00	−0.72	0.10	0.00	5.88	889.08
AAFPAPW	0.96	Non-Toxin	0.23	0.50	0.50	0.59	0.00	−1.06	0.14	0.00	5.88	758.95
GGGGIGGGGFGGF	0.96	Non-Toxin	0.27	0.68	0.68	0.47	0.00	−0.52	0.00	0.00	5.88	996.26
GFGGGGFGGGF	0.95	Non-Toxin	0.28	0.69	0.69	0.47	0.00	−0.68	0.00	0.00	5.88	916.13
GGGAGFGGGF	0.95	Non-Toxin	0.26	0.67	0.67	0.46	0.00	−0.55	0.00	0.00	5.88	782.96
GGGGFGGFG	0.95	Non-Toxin	0.26	0.68	0.68	0.31	0.00	−0.56	0.00	0.00	5.88	711.87
GGPGGPGFPL	0.95	Non-Toxin	0.17	0.57	0.57	−0.02	0.00	−0.43	0.00	0.00	5.88	855.11
SGGFGGGF	0.95	Non-Toxin	0.22	0.67	0.67	0.35	0.00	−0.59	0.12	0.00	5.88	684.82
GGGFGGGF	0.94	Non-Toxin	0.27	0.69	0.69	0.40	0.00	−0.62	0.00	0.00	5.88	654.80
GGFGGGF	0.94	Non-Toxin	0.29	0.69	0.69	0.51	0.00	−0.71	0.00	0.00	5.88	597.73
GGGFGSGGGF	0.94	Non-Toxin	0.21	0.67	0.67	0.20	0.00	−0.47	0.10	0.00	5.88	798.96
SFGGGGF	0.94	Non-Toxin	0.23	0.66	0.66	0.46	0.00	−0.67	0.14	0.00	5.88	627.75
GSGGGGFGGGGF	0.94	Non-Toxin	0.20	0.67	0.67	0.10	0.00	−0.39	0.08	0.00	5.88	913.10
GDGPLFGFT	0.94	Non-Toxin	0.14	0.62	0.62	0.27	0.00	−0.47	0.22	−1.00	3.80	910.12
NQPFFGG	0.93	Non-Toxin	0.02	0.65	0.65	−0.54	0.18	−0.66	0.57	0.00	5.88	765.92
GAGLGGGFGGGF	0.93	Non-Toxin	0.27	0.66	0.66	0.67	0.00	−0.61	0.00	0.00	5.88	953.21
GGSGFGSGGGF	0.93	Non-Toxin	0.17	0.66	0.66	0.11	0.00	−0.40	0.18	0.00	5.88	886.05
SSSGGFGGGF	0.92	Non-Toxin	0.12	0.64	0.64	0.12	0.00	−0.41	0.30	0.00	5.88	859.00
GGGFGGDGGLL	0.92	Non-Toxin	0.19	0.66	0.66	0.37	0.00	−0.28	0.09	−1.00	3.80	906.14
GFGGGGIGGGGF	0.92	Non-Toxin	0.28	0.68	0.68	0.54	0.00	−0.57	0.00	0.00	5.88	939.19
GDPAYPGGPL	0.91	Non-Toxin	0.04	0.56	0.56	−0.52	0.00	−0.16	0.20	−1.00	3.80	943.16
GGPGGFGPG	0.91	Non-Toxin	0.16	0.61	0.61	−0.31	0.00	−0.28	0.00	0.00	5.88	701.87
SGPDIGGF	0.91	Non-Toxin	0.10	0.64	0.64	0.02	0.00	−0.13	0.25	−1.00	3.80	748.90
AFDFIIR	0.91	Non-Toxin	0.06	0.68	0.68	1.20	0.35	−0.44	0.71	0.00	6.19	881.13
IDNIFRF	0.90	Non-Toxin	−0.06	0.71	0.71	0.44	0.35	−0.34	1.00	0.00	6.19	924.16
FADLHPF	0.90	Non-Toxin	0.12	0.51	0.51	0.41	0.21	−0.69	0.29	−0.50	5.09	846.04
GGGGSFGGGSFG	0.89	Non-Toxin	0.16	0.66	0.66	0.07	0.00	−0.37	0.17	0.00	5.88	943.12
GSPFTFA	0.89	Non-Toxin	0.16	0.57	0.57	0.56	0.00	−0.80	0.29	0.00	5.88	725.88
GGGYGGGGFGGG	0.89	Non-Toxin	0.19	0.68	0.68	−0.21	0.00	−0.40	0.08	0.00	5.88	899.08
QWDQGYF	0.89	Non-Toxin	−0.13	0.67	0.67	−1.47	0.36	−0.69	1.00	−1.00	3.80	943.08
SSSFGSGF	0.89	Non-Toxin	0.06	0.61	0.61	0.20	0.00	−0.47	0.50	0.00	5.88	774.88
GGGSFGGGSF	0.89	Non-Toxin	0.17	0.65	0.65	0.16	0.00	−0.44	0.20	0.00	5.88	828.98
AIDHPGFGL	0.88	Non-Toxin	0.14	0.55	0.55	0.42	0.16	−0.46	0.22	−0.50	5.09	926.17
GGGGIGGGGF	0.88	Non-Toxin	0.26	0.68	0.68	0.41	0.00	−0.43	0.00	0.00	5.88	734.93
GSPWYGPD	0.88	Non-Toxin	−0.05	0.57	0.57	−1.31	0.00	−0.30	0.50	−1.00	3.80	878.01
AWDGPALL	0.88	Non-Toxin	0.16	0.55	0.55	0.60	0.00	−0.62	0.25	−1.00	3.80	842.07
QHPALFL	0.88	Non-Toxin	0.11	0.47	0.47	0.56	0.39	−0.99	0.43	0.50	7.10	825.08
AFDFIIRN	0.87	Non-Toxin	−0.02	0.69	0.69	0.61	0.31	−0.36	0.88	0.00	6.19	995.25
SFYGDPL	0.87	Non-Toxin	0.04	0.61	0.61	−0.14	0.00	−0.47	0.43	−1.00	3.80	797.95
AIFYGPY	0.87	Non-Toxin	0.25	0.62	0.62	0.64	0.00	−1.34	0.29	0.00	5.87	830.04
HGGDFVLF	0.87	Non-Toxin	0.19	0.59	0.59	0.76	0.18	−0.72	0.25	−0.50	5.09	891.11
VDPSNFF	0.87	Non-Toxin	0.01	0.64	0.64	0.06	0.00	−0.43	0.57	−1.00	3.80	824.97
NSPFEFR	0.86	Non-Toxin	−0.30	0.63	0.63	−1.19	0.53	0.21	1.14	0.00	6.36	896.05
NQPFFGGV	0.86	Non-Toxin	0.08	0.66	0.66	0.05	0.16	−0.76	0.50	0.00	5.88	865.07
LDAVFFPR	0.86	Non-Toxin	0.00	0.62	0.62	0.72	0.31	−0.35	0.62	0.00	6.19	964.23
IAPSAGFL	0.86	Non-Toxin	0.28	0.57	0.57	1.49	0.00	−0.85	0.12	0.00	5.88	775.02
GGGGFGGGSF	0.85	Non-Toxin	0.21	0.67	0.67	0.20	0.00	−0.47	0.10	0.00	5.88	798.96
HFYPIWE	0.85	Non-Toxin	0.09	0.52	0.52	−0.46	0.39	−1.07	0.57	−0.50	5.25	991.21
SDPFGNNLL	0.85	Non-Toxin	−0.06	0.62	0.62	−0.32	0.00	−0.27	0.67	−1.00	3.80	976.18
GGSFGGGSF	0.85	Non-Toxin	0.17	0.65	0.65	0.22	0.00	−0.49	0.22	0.00	5.88	771.91
DFDLRPL	0.84	Non-Toxin	−0.23	0.62	0.62	−0.39	0.35	0.41	0.86	−1.00	4.21	875.08
HPGFVIGPL	0.84	Non-Toxin	0.24	0.52	0.52	0.90	0.16	−0.90	0.11	0.50	7.10	936.26
YNSPFEF	0.84	Non-Toxin	−0.05	0.63	0.63	−0.73	0.18	−0.54	0.71	−1.00	4.00	903.04
NYPAWGY	0.84	Non-Toxin	0.02	0.60	0.60	−1.03	0.00	−1.19	0.71	0.00	5.87	870.02
SGPAFNAGR	0.84	Non-Toxin	−0.14	0.60	0.60	−0.53	0.27	0.00	0.78	1.00	10.11	876.05
GGGSGGGFGGGY	0.83	Non-Toxin	0.15	0.67	0.67	−0.24	0.00	−0.38	0.17	0.00	5.88	929.10
SDPFGNNL	0.83	Non-Toxin	−0.13	0.64	0.64	−0.84	0.00	−0.08	0.75	−1.00	3.80	863.00
SWPVPGTL	0.83	Non-Toxin	0.13	0.52	0.52	0.25	0.00	−0.85	0.38	0.00	5.88	856.10
SGLWNWQ	0.83	Non-Toxin	−0.02	0.60	0.60	−0.89	0.18	−1.13	1.00	0.00	5.88	890.07
SLPAYAF	0.83	Non-Toxin	0.19	0.55	0.55	0.93	0.00	−1.04	0.29	0.00	5.88	767.96
VWHMPAL	0.83	Non-Toxin	0.21	0.48	0.48	0.86	0.21	−1.29	0.29	0.50	7.10	853.15
NGDPFIG	0.83	Non-Toxin	0.03	0.66	0.66	−0.30	0.00	−0.16	0.43	−1.00	3.80	718.86
SFGGGSF	0.83	Non-Toxin	0.17	0.64	0.64	0.40	0.00	−0.63	0.29	0.00	5.88	657.77
SPEGRLF	0.83	Non-Toxin	−0.20	0.59	0.59	−0.60	0.53	0.29	0.86	0.00	6.36	804.99
ASFGWVN	0.83	Non-Toxin	0.15	0.63	0.63	0.46	0.00	−1.06	0.57	0.00	5.88	779.94
GGGGFGGGGY	0.83	Non-Toxin	0.19	0.68	0.68	−0.17	0.00	−0.48	0.10	0.00	5.88	784.94
GFGGGAGGGF	0.83	Non-Toxin	0.26	0.67	0.67	0.46	0.00	−0.55	0.00	0.00	5.88	782.96
GLYGPGIW	0.82	Non-Toxin	0.26	0.60	0.60	0.41	0.00	−1.16	0.25	0.00	5.88	862.12
SDPAFRPH	0.82	Non-Toxin	−0.30	0.49	0.49	−1.33	0.49	0.35	0.88	0.50	7.10	926.09
VRPGSFF	0.82	Non-Toxin	−0.02	0.62	0.62	0.36	0.35	−0.46	0.71	1.00	10.11	809.02
NWDGWYT	0.82	Non-Toxin	−0.09	0.63	0.63	−1.60	0.00	−0.90	1.00	−1.00	3.80	941.06
GGSGGGFG	0.82	Non-Toxin	0.16	0.66	0.66	−0.05	0.00	−0.28	0.12	0.00	5.88	594.70
FDFIIRN	0.82	Non-Toxin	−0.06	0.71	0.71	0.44	0.35	−0.34	1.00	0.00	6.19	924.16
GHPWGNAPG	0.81	Non-Toxin	−0.01	0.50	0.50	−1.13	0.16	−0.47	0.44	0.50	7.10	892.07
LSPSTPSFF	0.81	Non-Toxin	0.07	0.53	0.53	0.34	0.00	−0.70	0.44	0.00	5.88	982.21
SANAGYRF	0.81	Non-Toxin	−0.17	0.64	0.64	−0.51	0.31	−0.29	1.00	1.00	9.10	885.04
NFNPGLY	0.81	Non-Toxin	0.00	0.64	0.64	−0.53	0.00	−0.89	0.71	0.00	5.88	824.00
WAIFRPQ	0.81	Non-Toxin	−0.08	0.59	0.59	−0.20	0.53	−0.71	1.00	1.00	10.11	917.17
WDQGYFQ	0.81	Non-Toxin	−0.13	0.67	0.67	−1.47	0.36	−0.69	1.00	−1.00	3.80	943.08
GADEGPVFF	0.81	Non-Toxin	0.10	0.64	0.64	0.24	0.14	−0.11	0.22	−2.00	3.67	938.12
GGGAGGGDGGIL	0.80	Non-Toxin	0.17	0.66	0.66	0.28	0.00	−0.09	0.08	−1.00	3.80	887.11
QSFYGDPL	0.80	Non-Toxin	−0.05	0.62	0.62	−0.56	0.16	−0.39	0.62	−1.00	3.80	926.10
SAPAFIQL	0.80	Non-Toxin	0.17	0.57	0.57	1.10	0.16	−0.82	0.38	0.00	5.88	846.10
SSKGSLGGGF	0.80	Non-Toxin	−0.01	0.62	0.62	−0.13	0.37	−0.04	0.50	1.00	9.11	896.11
SGSPANF	0.80	Non-Toxin	−0.03	0.58	0.58	−0.36	0.00	−0.31	0.57	0.00	5.88	678.78
APAFIQL	0.80	Non-Toxin	0.23	0.57	0.57	1.37	0.18	−0.99	0.29	0.00	5.88	759.01
GSGFGGGY	0.80	Non-Toxin	0.15	0.67	0.67	−0.16	0.00	−0.56	0.25	0.00	5.88	700.82
DYSLWIR	0.80	Non-Toxin	−0.16	0.63	0.63	−0.39	0.35	−0.43	1.14	0.00	6.19	952.17
AGDIGWRH	0.80	Non-Toxin	−0.15	0.57	0.57	−0.82	0.49	−0.03	0.88	0.50	7.10	911.10
IGGPGGNPF	0.80	Non-Toxin	0.13	0.62	0.62	−0.11	0.00	−0.46	0.22	0.00	5.88	815.03
GAPDGFDFE	0.80	Non-Toxin	−0.04	0.65	0.65	−0.61	0.14	0.39	0.33	−3.00	3.50	954.07
FYRVFPN	0.79	Non-Toxin	−0.10	0.66	0.66	−0.16	0.35	−0.80	1.00	1.00	9.10	942.17
SGDGAGGF	0.79	Non-Toxin	0.07	0.65	0.65	−0.16	0.00	0.04	0.25	−1.00	3.80	666.75
GPWTTNPL	0.79	Non-Toxin	−0.01	0.53	0.53	−0.70	0.00	−0.72	0.62	0.00	5.88	885.10
SGGYGGL	0.79	Non-Toxin	0.13	0.64	0.64	0.01	0.00	−0.54	0.29	0.00	5.88	609.74
AAPWGSAL	0.79	Non-Toxin	0.18	0.52	0.52	0.69	0.00	−0.80	0.25	0.00	5.88	771.97
DSSGVFSPF	0.79	Non-Toxin	0.04	0.61	0.61	0.21	0.00	−0.29	0.44	−1.00	3.80	942.10
ITHPLPF	0.79	Non-Toxin	0.16	0.45	0.45	0.57	0.21	−1.00	0.29	0.50	7.10	824.09
AWPVQGL	0.79	Non-Toxin	0.16	0.57	0.57	0.49	0.18	−1.00	0.43	0.00	5.88	770.00
SQPFFVA	0.79	Non-Toxin	0.14	0.60	0.60	0.81	0.18	−0.93	0.43	0.00	5.88	794.99

^1^ The likelihood of the peptides as bioactive was evaluated by PeptideRanker (http://bioware.ucd.ie/~compass/biowareweb, (accessed on 4 June 2025)), a server to predict bioactive peptides based on an N-to-1 neural network, by calculating scores ranging from 0 to 1. Higher scores indicated the greater the likelihood of the peptide being bioactive. ^2^ Peptides were subjected to calculation via https://webs.iiitd.edu.in/raghava/toxinpred/design.php/ (accessed on 4 June 2025), where the hydrophobicity, steric hinderance, and amphipathicity were calculated.

**Table 2:** Characterization of the most bioactive peptides from the peptidome according to the PeptideRanker prediction tool, identified in OLPH15A, based on in silico analyses in relation to the antimicrobial activity.

	CAMPR3 ^3^; Results with:							AMPfun ^4^						Antimicrobial Peptide Scanner vr.2 ^5^		Macrel ^6^
	Support Vector Machine (SVM) Classifier		Random Forest Classifier		Artificial Neural Network Classifier	Results with Discriminant Analysis Classifier		AMP	Anti-Parasitic	Anti-viral	Anti-fungal	Targeting Gram + Bacteria	Targeting Gram—Bacteria			AMP Probab
SGFGGFGGGF	AMP	0.82	NAMP	0.23	AMP	NAMP	0.35	0.91	0.42	0.43	0.63	0.65	0.76	AMP	0.91	0.26
AAFPAPW	NAMP	0.01	NAMP	0.47	AMP	NAMP	0.15	0.99	0.33	0.86	0.55	0.67	0.70	AMP *	0.78	0.15
GGGGIGGGGFGGF	AMP	0.77	NAMP	0.42	AMP	AMP	0.67	0.90	0.41	0.44	0.46	0.69	0.66	AMP	0.87	0.48
GFGGGGFGGGF	AMP	0.83	NAMP	0.29	AMP	AMP	0.55	0.89	0.47	0.47	0.58	0.70	0.76	AMP	0.86	0.47
GGGAGFGGGF	NAMP	0.04	NAMP	0.27	AMP	AMP	0.71	0.98	0.40	0.35	0.66	0.74	0.83	AMP	0.79	0.44
GGGGFGGFG	AMP	0.73	NAMP	0.29	AMP	NAMP	0.27	0.91	0.49	0.47	0.62	0.70	0.73	AMP *	0.88	0.43
GGPGGPGFPL	NAMP	0.00	NAMP	0.48	AMP	AMP	0.60	0.97	0.23	0.63	0.58	0.65	0.70	AMP	0.85	0.25
SGGFGGGF	AMP	0.87	NAMP	0.24	AMP	NAMP	0.13	0.90	0.40	0.43	0.64	0.68	0.73	AMP *	0.78	0.18
GGGFGGGF	AMP	0.74	NAMP	0.27	AMP	NAMP	0.21	0.90	0.42	0.47	0.63	0.68	0.74	AMP *	0.90	0.42
GGFGGGF	AMP	0.71	NAMP	0.28	AMP	NAMP	0.17	0.91	0.46	0.47	0.65	0.68	0.73	AMP *	0.86	0.42
GGGFGSGGGF	AMP	0.84	NAMP	0.26	AMP	NAMP	0.47	0.88	0.47	0.44	0.62	0.73	0.71	AMP	0.79	0.26
SFGGGGF	AMP	0.68	NAMP	0.24	AMP	NAMP	0.10	0.88	0.46	0.41	0.61	0.67	0.72	AMP *	0.68	0.16
GSGGGGFGGGGF	AMP	0.86	NAMP	0.31	AMP	AMP	0.57	0.91	0.41	0.42	0.60	0.64	0.74	AMP	0.73	0.31
GDGPLFGFT	NAMP	0.01	NAMP	0.46	AMP	NAMP	0.05	0.97	0.10	0.59	0.24	0.48	0.48	Non-AMP *	0.41	0.21
NQPFFGG	AMP	0.96	NAMP	0.36	AMP	NAMP	0.25	1.00	0.41	0.43	0.67	0.66	0.65	AMP *	0.65	0.03
GAGLGGGFGGGF	NAMP	0.34	NAMP	0.34	AMP	AMP	0.84	0.96	0.37	0.33	0.60	0.71	0.70	AMP	0.95	0.42
GGSGFGSGGGF	AMP	0.93	NAMP	0.26	AMP	NAMP	0.33	0.90	0.47	0.46	0.61	0.68	0.71	AMP	0.64	0.28
SSSGGFGGGF	AMP	0.98	NAMP	0.24	NAMP	NAMP	0.06	0.90	0.42	0.43	0.58	0.69	0.68	AMP	0.77	0.32
GGGFGGDGGLL	NAMP	0.00	NAMP	0.25	AMP	NAMP	0.16	0.93	0.15	0.44	0.64	0.66	0.61	Non-AMP	0.38	0.25
GFGGGGIGGGGF	AMP	0.75	NAMP	0.38	AMP	AMP	0.56	0.92	0.42	0.45	0.50	0.73	0.71	AMP	0.70	0.46
GDPAYPGGPL	NAMP	0.00	NAMP	0.37	AMP	NAMP	0.06	0.96	0.13	0.63	0.50	0.52	0.45	Non-AMP	0.41	0.24
GGPGGFGPG	AMP	0.74	NAMP	0.33	AMP	NAMP	0.34	0.91	0.35	0.50	0.62	0.61	0.76	AMP *	0.86	0.29
SGPDIGGF	NAMP	0.05	NAMP	0.30	NAMP	NAMP	0.00	0.89	0.56	0.43	0.56	0.58	0.55	Non-AMP *	0.16	0.12
AFDFIIR	NAMP	0.04	NAMP	0.39	AMP	NAMP	0.07	0.94	0.26	0.79	0.61	0.53	0.53	Non-AMP *	0.34	0.27
IDNIFRF	NAMP	0.00	NAMP	0.42	AMP	NAMP	0.11	0.92	0.09	0.63	0.61	0.57	0.31	Non-AMP *	0.34	0.20
FADLHPF	NAMP	0.09	NAMP	0.39	AMP	NAMP	0.01	0.97	0.25	0.77	0.54	0.48	0.44	Non-AMP *	0.30	0.11
GGGGSFGGGSFG	AMP	0.92	NAMP	0.33	AMP	NAMP	0.39	0.87	0.51	0.45	0.61	0.64	0.71	AMP	0.79	0.26
GSPFTFA	NAMP	0.06	NAMP	0.45	AMP	NAMP	0.16	0.99	0.34	0.74	0.62	0.68	0.74	AMP *	0.68	0.07
GGGYGGGGFGGG	AMP	0.55	NAMP	0.39	AMP	NAMP	0.28	0.90	0.55	0.45	0.61	0.70	0.64	AMP	0.75	0.27
QWDQGYF	NAMP	0.01	NAMP	0.33	NAMP	NAMP	0.02	0.95	0.11	0.91	0.40	0.53	0.37	Non-AMP *	0.26	0.07
SSSFGSGF	AMP	1.00	NAMP	0.21	NAMP	NAMP	0.01	0.87	0.42	0.43	0.61	0.66	0.62	AMP *	0.84	0.30
GGGSFGGGSF	AMP	0.94	NAMP	0.26	AMP	NAMP	0.28	0.88	0.42	0.45	0.61	0.73	0.74	AMP	0.78	0.23
AIDHPGFGL	NAMP	0.11	NAMP	0.44	AMP	NAMP	0.11	0.99	0.07	0.47	0.64	0.52	0.49	Non-AMP *	0.28	0.09
GGGGIGGGGF	NAMP	0.22	NAMP	0.34	AMP	NAMP	0.25	0.90	0.64	0.47	0.47	0.70	0.68	AMP	0.70	0.45
GSPWYGPD	NAMP	0.00	NAMP	0.32	NAMP	NAMP	0.00	0.84	0.33	0.67	0.49	0.54	0.55	Non-AMP *	0.07	0.23
AWDGPALL	NAMP	0.00	NAMP	0.41	AMP	NAMP	0.10	0.98	0.15	0.84	0.40	0.52	0.48	AMP *	0.53	0.10
QHPALFL	NAMP	0.00	NAMP	0.45	AMP	NAMP	0.25	0.99	0.26	0.70	0.61	0.68	0.46	AMP *	0.51	0.21
AFDFIIRN	NAMP	0.06	NAMP	0.32	AMP	NAMP	0.09	0.91	0.22	0.83	0.62	0.54	0.32	Non-AMP *	0.34	0.18
SFYGDPL	AMP	1.00	NAMP	0.38	NAMP	NAMP	0.00	0.96	0.17	0.73	0.61	0.52	0.51	Non-AMP *	0.29	0.10
AIFYGPY	NAMP	0.00	NAMP	0.28	AMP	NAMP	0.10	0.94	0.30	0.63	0.58	0.62	0.64	Non-AMP *	0.48	0.03
HGGDFVLF	NAMP	0.02	NAMP	0.39	NAMP	NAMP	0.01	0.98	0.19	0.63	0.53	0.53	0.59	Non-AMP *	0.03	0.17
VDPSNFF	NAMP	0.27	NAMP	0.39	NAMP	NAMP	0.00	0.87	0.39	0.82	0.44	0.58	0.48	Non-AMP *	0.24	0.07
NSPFEFR	AMP	0.98	NAMP	0.41	NAMP	NAMP	0.00	0.97	0.21	0.58	0.71	0.59	0.51	Non-AMP *	0.46	0.31
NQPFFGGV	NAMP	0.42	NAMP	0.30	NAMP	NAMP	0.14	0.99	0.33	0.45	0.55	0.66	0.55	AMP *	0.62	0.04
LDAVFFPR	NAMP	0.00	NAMP	0.43	AMP	NAMP	0.02	0.98	0.36	0.66	0.59	0.47	0.45	Non-AMP *	0.31	0.05
IAPSAGFL	NAMP	0.06	NAMP	0.31	AMP	AMP	0.81	0.98	0.42	0.63	0.60	0.75	0.70	Non-AMP *	0.37	0.09
GGGGFGGGSF	AMP	0.69	NAMP	0.28	AMP	NAMP	0.47	0.90	0.44	0.37	0.63	0.73	0.69	AMP	0.79	0.29
HFYPIWE	NAMP	0.00	NAMP	0.27	NAMP	NAMP	0.00	0.93	0.39	0.89	0.54	0.47	0.44	Non-AMP *	0.22	0.12
SDPFGNNLL	NAMP	0.00	NAMP	0.38	NAMP	NAMP	0.02	0.98	0.15	0.51	0.54	0.53	0.43	Non-AMP *	0.23	0.02
GGSFGGGSF	AMP	0.94	NAMP	0.25	AMP	NAMP	0.19	0.90	0.46	0.45	0.65	0.69	0.73	AMP *	0.76	0.16
DFDLRPL	NAMP	0.00	NAMP	0.34	NAMP	NAMP	0.00	0.99	0.36	0.83	0.46	0.48	0.53	Non-AMP *	0.27	0.08
HPGFVIGPL	NAMP	0.07	AMP	0.56	NAMP	NAMP	0.21	0.98	0.27	0.53	0.58	0.73	0.68	AMP *	0.84	0.11
YNSPFEF	AMP	1.00	NAMP	0.40	NAMP	NAMP	0.01	0.98	0.21	0.67	0.62	0.50	0.63	Non-AMP *	0.24	0.22
NYPAWGY	NAMP	0.01	NAMP	0.27	NAMP	NAMP	0.01	1.00	0.04	0.83	0.53	0.65	0.52	AMP *	0.56	0.07
SGPAFNAGR	NAMP	0.01	NAMP	0.27	AMP	NAMP	0.05	0.98	0.07	0.60	0.73	0.73	0.76	Non-AMP *	0.46	0.09
GGGSGGGFGGGY	AMP	0.83	NAMP	0.40	AMP	NAMP	0.16	0.90	0.48	0.42	0.57	0.61	0.65	AMP	0.77	0.28
SDPFGNNL	NAMP	0.01	NAMP	0.40	NAMP	NAMP	0.02	0.97	0.20	0.57	0.58	0.57	0.52	Non-AMP *	0.44	0.10
SWPVPGTL	NAMP	0.00	NAMP	0.23	AMP	NAMP	0.01	0.98	0.26	0.75	0.39	0.66	0.74	AMP *	0.87	0.04
SGLWNWQ	NAMP	0.00	NAMP	0.26	AMP	NAMP	0.07	0.97	0.09	0.78	0.59	0.69	0.69	AMP *	0.94	0.14
SLPAYAF	NAMP	0.07	NAMP	0.25	NAMP	NAMP	0.05	0.98	0.21	0.91	0.64	0.64	0.62	AMP *	0.71	0.09
VWHMPAL	NAMP	0.00	AMP	0.51	AMP	NAMP	0.02	0.95	0.34	0.90	0.42	0.58	0.62	AMP *	0.67	0.17
NGDPFIG	NAMP	0.00	NAMP	0.43	AMP	NAMP	0.00	0.92	0.34	0.54	0.53	0.47	0.42	Non-AMP *	0.43	0.07
SFGGGSF	AMP	0.84	NAMP	0.22	NAMP	NAMP	0.04	0.89	0.46	0.44	0.62	0.68	0.71	AMP *	0.76	0.15
SPEGRLF	NAMP	0.07	NAMP	0.42	NAMP	NAMP	0.00	0.95	0.16	0.78	0.63	0.66	0.48	Non-AMP *	0.43	0.18
ASFGWVN	NAMP	0.04	NAMP	0.25	AMP	NAMP	0.03	0.99	0.21	0.54	0.57	0.63	0.69	AMP *	0.59	0.14
GGGGFGGGGY	AMP	0.55	NAMP	0.32	AMP	NAMP	0.18	0.92	0.47	0.48	0.62	0.69	0.65	AMP	0.68	0.29
GFGGGAGGGF	NAMP	0.07	NAMP	0.26	AMP	AMP	0.71	0.96	0.44	0.33	0.71	0.73	0.80	AMP	0.85	0.44
GLYGPGIW	NAMP	0.02	NAMP	0.32	AMP	NAMP	0.09	0.94	0.29	0.68	0.40	0.64	0.63	AMP *	0.56	0.08
SDPAFRPH	NAMP	0.33	NAMP	0.41	NAMP	NAMP	0.08	0.95	0.10	0.78	0.44	0.48	0.37	Non-AMP *	0.27	0.13
VRPGSFF	NAMP	0.11	NAMP	0.36	NAMP	NAMP	0.03	0.98	0.30	0.68	0.71	0.74	0.64	AMP *	0.79	0.08
NWDGWYT	NAMP	0.00	NAMP	0.33	NAMP	NAMP	0.00	0.90	0.39	0.86	0.31	0.48	0.39	Non-AMP *	0.47	0.06
GGSGGGFG	AMP	0.62	NAMP	0.29	AMP	NAMP	0.14	0.86	0.56	0.46	0.60	0.61	0.67	AMP *	0.74	0.23
FDFIIRN	NAMP	0.00	NAMP	0.37	AMP	NAMP	0.11	0.90	0.25	0.81	0.59	0.52	0.40	Non-AMP *	0.43	0.25
GHPWGNAPG	NAMP	0.12	NAMP	0.25	AMP	NAMP	0.05	0.99	0.03	0.82	0.49	0.72	0.72	AMP *	0.78	0.13
LSPSTPSFF	AMP	0.79	NAMP	0.34	NAMP	NAMP	0.05	0.97	0.59	0.72	0.48	0.66	0.70	AMP *	0.50	0.15
SANAGYRF	NAMP	0.01	NAMP	0.32	AMP	NAMP	0.02	0.99	0.12	0.59	0.69	0.63	0.63	Non-AMP *	0.47	0.11
NFNPGLY	NAMP	0.00	NAMP	0.25	AMP	NAMP	0.00	0.96	0.21	0.83	0.67	0.67	0.55	AMP *	0.64	0.04
WAIFRPQ	NAMP	0.02	NAMP	0.33	AMP	NAMP	0.28	0.98	0.19	0.63	0.58	0.62	0.64	AMP *	0.61	0.08
WDQGYFQ	NAMP	0.00	NAMP	0.33	NAMP	NAMP	0.05	0.97	0.06	0.86	0.38	0.56	0.46	Non-AMP *	0.42	0.03
GADEGPVFF	NAMP	0.03	NAMP	0.41	AMP	NAMP	0.02	0.94	0.41	0.54	0.53	0.49	0.44	Non-AMP *	0.22	0.20
GGGAGGGDGGIL	NAMP	0.00	NAMP	0.38	AMP	NAMP	0.05	0.85	0.18	0.31	0.50	0.56	0.60	Non-AMP	0.07	0.21
QSFYGDPL	NAMP	0.20	NAMP	0.25	NAMP	NAMP	0.00	0.98	0.08	0.74	0.55	0.55	0.47	Non-AMP *	0.16	0.05
SAPAFIQL	NAMP	0.00	NAMP	0.28	AMP	NAMP	0.24	0.98	0.36	0.79	0.66	0.74	0.54	Non-AMP *	0.23	0.08
SSKGSLGGGF	NAMP	0.34	NAMP	0.33	AMP	NAMP	0.42	1.00	0.11	0.43	0.57	0.69	0.72	AMP	0.80	0.18
SGSPANF	NAMP	0.07	NAMP	0.22	AMP	NAMP	0.02	0.98	0.50	0.69	0.60	0.58	0.53	AMP *	0.64	0.17
APAFIQL	NAMP	0.00	NAMP	0.44	AMP	AMP	0.78	0.99	0.37	0.79	0.72	0.72	0.57	Non-AMP *	0.42	0.30
GSGFGGGY	AMP	0.74	NAMP	0.27	NAMP	NAMP	0.03	0.80	0.49	0.47	0.65	0.66	0.66	AMP *	0.64	0.12
DYSLWIR	AMP	0.88	NAMP	0.40	NAMP	NAMP	0.01	0.99	0.09	0.74	0.56	0.49	0.55	Non-AMP *	0.11	0.05
AGDIGWRH	NAMP	0.00	NAMP	0.36	AMP	NAMP	0.01	0.98	0.26	0.48	0.52	0.53	0.47	Non-AMP *	0.17	0.22
IGGPGGNPF	AMP	0.87	NAMP	0.28	AMP	NAMP	0.16	0.94	0.60	0.48	0.64	0.68	0.69	AMP *	0.85	0.11
GAPDGFDFE	NAMP	0.00	NAMP	0.34	NAMP	NAMP	0.02	0.98	0.09	0.59	0.45	0.48	0.38	Non-AMP *	0.03	0.08
FYRVFPN	NAMP	0.00	NAMP	0.35	AMP	NAMP	0.11	0.97	0.47	0.49	0.61	0.62	0.70	AMP *	0.65	0.11
SGDGAGGF	NAMP	0.00	NAMP	0.32	AMP	NAMP	0.04	0.94	0.61	0.43	0.61	0.50	0.47	Non-AMP *	0.16	0.10
GPWTTNPL	NAMP	0.00	NAMP	0.29	AMP	NAMP	0.02	0.97	0.08	0.88	0.47	0.64	0.60	AMP *	0.89	0.22
SGGYGGL	NAMP	0.00	NAMP	0.22	AMP	NAMP	0.01	0.99	0.29	0.55	0.71	0.75	0.67	AMP *	0.56	0.15
AAPWGSAL	NAMP	0.00	NAMP	0.26	AMP	NAMP	0.18	0.96	0.36	0.78	0.41	0.73	0.72	AMP *	0.60	0.12
DSSGVFSPF	AMP	0.99	NAMP	0.43	NAMP	NAMP	0.01	0.92	0.31	0.64	0.50	0.53	0.43	Non-AMP *	0.42	0.15
ITHPLPF	NAMP	0.07	NAMP	0.31	AMP	NAMP	0.17	0.98	0.22	0.68	0.56	0.63	0.67	AMP *	0.67	0.12
AWPVQGL	NAMP	0.00	NAMP	0.30	AMP	NAMP	0.11	0.98	0.17	0.82	0.59	0.68	0.59	AMP *	0.62	0.04
SQPFFVA	AMP	0.68	NAMP	0.44	NAMP	NAMP	0.12	0.97	0.41	0.75	0.55	0.67	0.62	AMP *	0.52	0.12

The antimicrobial activity, general and specific to bacteria, viruses, and fungi, was evaluated using different tools ^3^ CAMPR3 (http://www.camp3.bicnirrh.res.in/ (accessed on 11 June 2025), ^4^ AMPfun (http://fdblab.csie.ncu.edu.tw/AMPfun/index.html (accessed on 11 June 2025)), ^5^ Antimicrobial Peptide Scanner vr.2 (https://www.dveltri.com/ascan/v2/ascan.html (accessed on 11 June 2025)), ^6^ Macrel (https://big-data-biology.org/software/macrel (accessed on 11 June 2025)). * Those marked with an asterisk indicate internal prediction inconsistencies. Each tool might have a different threshold, but a higher score indicates the greater the likelihood of the peptide being bioactive.

**Table 3:** Molecular docking results, including the affinity of each peptide with bacterial target receptors, the cluster size, and the interaction (position, type, and distance).

Receptor	Peptide	Docking Results				
		Possible Best Result				
		Affinity (Kcal/mol)	Cluster Size	Interactions		
				Interaction Residues	Bond Distance (Å)	Type of Interaction Bond
3QDL	NYPAWGY	−14.2	34	Asn1–Ser30 (A)	3.38	*Carbon–Hydrogen Bond*
					2.70	*Conventional Hydrogen Bond*
				Asn1–Leu210 (A)	2.80	*Salt Bridge*
				Tyr2–Thr58 (A)	3.01	*Conventional Hydrogen Bond*
				Tyr2–Phe28 (A)	3.27	*Conventional Hydrogen Bond*
				Tyr2–Ser81 (A)	2.32	*Conventional Hydrogen Bond*
				Trp5–Glu27 (A)	2.94	*Conventional Hydrogen Bond*
				Trp5–Ser81 (A)	2.97	*Carbon–Hydrogen Bond*
				Trp5–Ala80 (A)	2.73	*Conventional Hydrogen Bond*
				Gly6–Lys60 (A)	2.63	*Conventional Hydrogen Bond*
				Tyr7–Asp59 (A)	3.00	*Pi-Anion*
	SSKGSLGGGF	−13.8	54	Ser1–Arg176 (A)	2.72	*Conventional Hydrogen Bond*
					5.53	*Unfavorable Positive-Positive*
				Ser1–Trp209 (A)	2.76	*Conventional Hydrogen Bond*
					3.24	
				Lys3–Leu210 (B)	4.63	*Alkyl*
				Lys3–Asp59 (A)	2.68	*Salt Bridge*
				Gly4–Thr58 (A)	3.20	*Conventional Hydrogen Bond*
				Ser5–Leu210 (B)	2.71	*Conventional Hydrogen Bond*
				Ser5–Thr58 (A)	2.76	*Carbon–Hydrogen Bond*
				Gly7–Phe28 (A)	2.80	*Conventional Hydrogen Bond*
					2.82	
				Gly7–Glu27 (A)	3.48	*Carbon–Hydrogen Bond*
				Gly8–Thr58 (A)	3.19	*Carbon–Hydrogen Bond*
				Gly9–Lys63 (A)	2.76	*Conventional Hydrogen Bond*
				Gly9–Ser81 (A)	2.92	*Conventional Hydrogen Bond*
				Gly9–Ala80 (A)	3.05	*Carbon–Hydrogen Bond*
				Phe10–Ser24 (A)	3.04	*Conventional Hydrogen Bond*
				Phe10–Lys78 (A)	4.83	*Pi-Alkyl*
4ASJ	NYPAWGY	−12.5	45	Asn1–Leu159 (A)	3.04	*Conventional Hydrogen Bond*
					3.72	*Carbon–Hydrogen Bond*
				Asn1–Arg204 (A)	2.93	*Conventional Hydrogen Bond*
				Asn1–Glu160 (A)	2.83	*Salt Bridge*
				Asn1–Glu149 (A)	5.32	*Attractive Charge*
				Tyr2–Glu161 (A)	2.66	*Conventional Hydrogen Bond*
				Tyr2–Arg204 (A)	2.54	*Conventional Hydrogen Bond*
				Ala4–Asp201 (A)	2.65	*Conventional Hydrogen Bond*
				Ala4–Ser192 (A)	2.76	*Carbon–Hydrogen Bond*
				Ala4–Pro193 (A)	3.18	*Carbon–Hydrogen Bond*
				Trp5–Asp201 (A)	2.70	*Conventional Hydrogen Bond*
					4.71	*Pi-Anion*
				Tyr7–Pro193 (A)	5.15	*Pi-Alkyl*
					2.70	*Conventional Hydrogen Bond*
				Tyr7–Pro191 (A)	3.73	*Carbon–Hydrogen Bond*
	SSKGSLGGGF	−15.3	37	Ser1–Glu165 (A)	2.35	*Conventional Hydrogen Bond*
					2.61	*Salt Bridge*
				Lys3–Glu165 (A)	3.15	*Conventional Hydrogen Bond*
					2.42	
				Lys3–Glu160 (A)	2.83	*Salt Bridge*
				Leu6–Pro163 (A)	5.38	*Alkyl*
				Leu6–Arg194 (A)	2.87	*Conventional Hydrogen Bond*
				Gly7–Glu161 (A)	2.96	*Conventional Hydrogen Bond*
				Gly7–Lys162 (A)	2.83	*Conventional Hydrogen Bond*
				Phe10–Glu143 (A)	4.40	*Pi-Anion*
				Phe10–Glu143 (A)	3.86	*Pi-Sigma*
4KQQ	NYPAWGY	−10.6	55	Asn1–Thr256 (A)	2.75	*Conventional Hydrogen Bond*
				Asn1–Pro433 (A)	2.28	*Conventional Hydrogen Bond*
				Tyr2–Thr437 (A)	3.16	*Conventional Hydrogen Bond*
				Tyr2–Ser435 (A)	3.83	*Pi-Donor Hydrogen Bond*
				Tyr2–Met436 (A)	3.95	*Carbon–Hydrogen Bond*
					5.91	*Pi-Sulfur*
				Tyr2–Leu381 (A)	5.41	*Pi-Alkyl*
				Ala4–Gly380 (A)	2.360	*Unfavorable Bump*
				Trp5–Pro433 (A)	5.11	*Pi-Alkyl*
				Trp5–Asp378 (A)	2.52	*Unfavorable Acceptor-Acceptor*
				Trp5–Ser431 (A)	2.56	*Conventional Hydrogen Bond*
				Trp5–Val446 (A)	3.89	*Pi-Sigma*
					5.42	*Pi-Alkyl*
				Gly6–Gly374 (A)	3.02	*Carbon–Hydrogen Bond*
				Tyr7–Val448 (A)	5.39	*Pi-Alkyl*
				Tyr7–Gln372 (A)	2.96	*Pi-Lone Pair*
	SSKGSLGGGF	−7.5	24	Ser1–His420 (A)	2.97	*Conventional Hydrogen Bond*
				Ser1–Thr379 (A)	3.63	*Carbon–Hydrogen Bond*
					2.68	*Conventional Hydrogen Bond*
				Ser1–Asp378 (A)	2.71	*Carbon–Hydrogen Bond*
				Ser2–Gln372 (A)	3.07	*Conventional Hydrogen Bond*
				Lys3–Gln372 (A)	2.24	*Conventional Hydrogen Bond*
				Lys3–Val448 (A)	4.95	*Alkyl*
				Lys3–Val446 (A)	3.88	*Alkyl*
				Lys3–Pro433 (A)	2.80	*Carbon–Hydrogen Bond*
				Ser5–Asp378 (A)	3.29	*Conventional Hydrogen Bond*
				Ser5–Gly380 (A)	2.32	*Unfavorable Bump*
				Leu6–Gly380 (A)	2.13	*Unfavorable Acceptor-Acceptor*
				Leu6–Leu381 (A)	4.49	*Alkyl*
				Leu6–Met436 (A)	4.89	*Alkyl*
				Gly9–Pro433 (A)	2.39	*Unfavorable Bump*

**Table 4:** Molecular docking results, including the affinity of each peptide with the COVID-19 main protease in the apo state, the cluster size, and the interaction (position, type, and distance).

Receptor	Peptide	Docking Results				
		Possible Best Result				
		Affinity (Kcal/mol)	Cluster Size	Interactions		
				Interaction Residues	Bond Distance (Å)	Type of Interaction Bond
7C2Q	QWDQGYF	−13.4	24	Gln1–Gln110 (B)	3.34	*Unfavorable Donor-Donor*
				Asp3–Tyr154 (B)	2.68	*Conventional Hydrogen Bond*
				Asp3–Ile152 (B)	3.02	*Conventional Hydrogen Bond*
				Asp3–Arg298 (B)	2.45	*Carbon–Hydrogen Bond*
				Tyr6–Arg298 (B)	2.86	*Conventional Hydrogen Bond*
				Tyr6–Ile152 (B)	4.62	*Pi-Alkyl*
				Tyr6–Pro9 (B)	5.48	*Pi-Alkyl*
				Tyr6–Phe8 (B)	5.13	*Pi-Pi T-shaped*
				Phe7–Ser123 (A)	4.12	*Conventional Hydrogen Bond*
	SLPAYAF	−10.9	54	Ser1–Val125 (A)	3.37	*Conventional Hydrogen Bond*
					2.54	
				Ser1–Lys5 (B)	3.09	*Conventional Hydrogen Bond*
				Ser1–Ala7 (B)	3.02	*Unfavorable Donor-Donor*
				Ser1–Gln127 (A)	2.91	*Conventional Hydrogen Bond*
				Leu2–Arg4 (A)	4.94	*Alkyl*
				Leu2–Lys5 (B)	3.09	*Alkyl*
				Pro3–Lys5 (A)	4.36	*Alkyl*
				Pro4-Phe3 (B)	2.26	*Unfavorable Bump*
					2.20	*Unfavorable Bump*
				Tyr5–Lys5 (B)	3.04	*Conventional Hydrogen Bond*
				Ala6–Phe291 (B)	5.26	*Pi-Alkyl*
				Phe7–Lys5 (B)	2.92	*Unfavorable Donor-Donor*
					2.50	*Conventional Hydrogen Bond*
				Phe7–Arg4 (A)	2.55	*Conventional Hydrogen Bond*

**Table 5:** Molecular docking results, including the affinity of each peptide with the lanosterol 14α-demethylase, the cluster size, and the interaction (position, type, and distance).

Receptor	Peptide	Docking Results				
		Possible Best Result				
		Affinity (Kcal/mol)	Cluster Size	Interactions		
				Interaction Residues	Bond Distance (Å)	Type of Interaction Bond
4LXJ	APAFIQL	−9.2	23	Ala1–Trp45 (A)	3.74	*Pi-Sigma*
					4.06	*Pi-Alkyl*
				Pro2–Tyr41 (A)	4.86	*Pi-Alkyl*
				Phe4–Trp65 (A)	2.93	*Carbon–Hydrogen Bond*
					4.93	*Pi-Pi Stacked*
				Gln6–Trp65 (A)	3.36	*Unfavourable Donor-Donor*
				Gln6–Arg98 (A)	2.94	*Unfavourable Donor-Donor*
				Gln6–Leu96 (A)	2.96	*Conventional Hydrogen Bond*
				Leu7–Gly97 (A)	2.75	*Conventional Hydrogen Bond*
				Leu7–Val99 (A)	5.15	*Alkyl*
	SGPAFNAGR	−9.9	34	Ser1–Pro64 (A)	2.95	*Carbon–Hydrogen Bond*
				Gly2–Pro64 (A)	2.83	*Conventional Hydrogen Bond*
				Pro3–Trp65 (A)	5.73	*Pi-Alkyl*
				Ala4–Trp65 (A)	4.43	*Pi-Alkyl*
				Asn6–Val242 (A)	2.55	*Conventional Hydrogen Bond*
				Asn6–Arg98 (A)	2.93	*Conventional Hydrogen Bond*
					5.75	
				Ala7–Trp65 (A)	4.61	*Pi-Alkyl*
				Gly8–Glu97 (A)	3.06	*Conventional Hydrogen Bond*
				Arg9–Glu97 (A)	2.78	*Conventional Hydrogen Bond*
				Arg9–Val99 (A)	4.43	*Alkyl*
				Arg9–Tyr404 (A)	5.44	*Pi-Alkyl*
				Arg9–Ala402 (A)	2.98	*Conventional Hydrogen Bond*

## Data Availability

Dataset is available on request from the authors.
